# Mast Cell Chymase/Mcpt4 Suppresses the Host Immune Response to *Plasmodium yoelii*, Limits Malaria-Associated Disruption of Intestinal Barrier Integrity and Reduces Parasite Transmission to *Anopheles stephensi*

**DOI:** 10.3389/fimmu.2022.801120

**Published:** 2022-01-27

**Authors:** Nora Céspedes, Erinn L. Donnelly, Casey Lowder, Gretchen Hansten, Delaney Wagers, Anna M. Briggs, Joseph Schauer, Lori Haapanen, Magnus Åbrink, Judy Van de Water, Shirley Luckhart

**Affiliations:** ^1^ Department of Entomology, Plant Pathology and Nematology, University of Idaho, Moscow, ID, United States; ^2^ Department of Biological Sciences, University of Idaho, Moscow, ID, United States; ^3^ Division of Rheumatology, Allergy and Clinical Immunology, University of California, Davis, Davis, CA, United States; ^4^ Section of Immunology, Department of Biomedical Sciences & Veterinary Public Health, Swedish University of Agricultural Sciences, Uppsala, Sweden

**Keywords:** malaria, bacteremia, mast cells, Mcpt4, chymase, *Plasmodium yoelii*, *Anopheles stephensi*, intestinal permeability

## Abstract

An increase in mast cells (MCs) and MCs mediators has been observed in malaria-associated bacteremia, however, the role of these granulocytes in malarial immunity is poorly understood. Herein, we studied the role of mouse MC protease (Mcpt) 4, an ortholog of human MC chymase, in malaria-induced bacteremia using *Mcpt4* knockout (*Mcpt4* ^-/-^) mice and *Mcpt4* ^+/+^ C57BL/6J controls, and the non-lethal mouse parasite *Plasmodium yoelii yoelii* 17XNL. Significantly lower parasitemia was observed in *Mcpt4* ^-/-^ mice compared with *Mcpt4* ^+/+^ controls by day 10 post infection (PI). Although bacterial 16S DNA levels in blood were not different between groups, increased intestinal permeability to FITC-dextran and altered ileal adherens junction E-cadherin were observed in *Mcpt4* ^-/-^ mice. Relative to infected *Mcpt4* ^+/+^ mice, ileal MC accumulation in *Mcpt4* ^-/-^ mice occurred two days earlier and IgE levels were higher by days 8-10 PI. Increased levels of circulating myeloperoxidase were observed at 6 and 10 days PI in *Mcpt4* ^+/+^ but not *Mcpt4* ^-/-^ mice, affirming a role for neutrophil activation that was not predictive of parasitemia or bacterial 16S copies in blood. In contrast, early increased plasma levels of TNF-α, IL-12p40 and IL-3 were observed in *Mcpt4* ^-/-^ mice, while levels of IL-2, IL-10 and MIP1β (CCL4) were increased over the same period in *Mcpt4* ^+/+^ mice, suggesting that the host response to infection was skewed toward a type-1 immune response in *Mcpt4* ^-/-^ mice and type-2 response in *Mcpt4* ^+/+^ mice. Spearman analysis revealed an early (day 4 PI) correlation of *Mcpt4* ^-/-^ parasitemia with TNF-α and IFN-γ, inflammatory cytokines known for their roles in pathogen clearance, a pattern that was observed in *Mcpt4* ^+/+^ mice much later (day 10 PI). Transmission success of *P. y. yoelii* 17XNL to *Anopheles stephensi* was significantly higher from infected *Mcpt4* ^-/-^ mice compared with infected *Mcpt4* ^+/+^ mice, suggesting that Mcpt4 also impacts transmissibility of sexual stage parasites. Together, these results suggest that early MCs activation and release of Mcpt4 suppresses the host immune response to *P. y. yoelii* 17XNL, perhaps *via* degradation of TNF-α and promotion of a type-2 immune response that concordantly protects epithelial barrier integrity, while limiting the systemic response to bacteremia and parasite transmissibility.

## Introduction

Malaria is a vector-borne parasitic disease caused by infection with protozoa of the genus *Plasmodium*. The disease is endemic in tropical and sub-tropical regions worldwide and affects about 45% of the global population. In 2019, there were an estimated of 229 million malaria cases and 409,000 deaths, mainly due to *Plasmodium falciparum*, with 67% of malaria deaths in children under 5 years (1). Numerous studies have indicated that children with acute falciparum malaria are predisposed to developing concomitant bacteremia, resulting in increased risk of mortality (2). In contrast, a lower prevalence of bacteremia has been reported in adults with severe malaria (3). However, as observed in children with malaria, adult bacteremic patients present with increased disease severity and have a greater risk of death than non-bacteremic patients regardless of parasitemia levels (4; 5).

Our studies with malaria in mouse and non-human primate models have demonstrated that intestinal permeability and bacteremia are functionally associated with increased intestinal infiltration of mast cells (MCs) or intestinal mastocytosis (6–8). MCs are rare granulocytes that can respond to and synthesize a large number of effectors including prostaglandin D2, cytokines, chemokines, heparin and histamine as well as a variety of proteases such as tryptases, carboxypeptidase A3 and chymases (9–11). In previous work, we demonstrated that L-arginine and L-citrulline supplementation reversed murine malaria-induced intestinal mastocytosis, significantly reduced bacteremia, and improved epithelial barrier morphology (6). Further, we showed that MC-deficient mice with malaria exhibited reduced gut permeability and bacteremia relative to controls, with histamine contributing to some but not all of this pathology (7), supporting the involvement of MCs in malaria-induced gut barrier disruption. In recent studies with the nonlethal mouse parasite *Plasmodium yoelii yoelii* 17XNL, we observed increased circulating MC protease-4 (Mcpt4) at 4 days post infection (PI) (10-fold above control) and 8 days PI (6-fold above control) and sharply increased numbers of intestinal MCs on those same days. Infection was also associated with rising levels of IgE and the MC-activating type-2 cytokines interleukin (IL)-3, -4, -5, -9, -10 and -13 as well as increased intestinal permeability and bacteremia through 10 days PI (8).

Mouse Mcpt4 is functionally homologous to human chymase and has been associated with MC-dependent regulation of intestinal epithelial cell migration and barrier function (12), the extravascular coagulation system (13), survival in models of sepsis and traumatic spinal cord injury and recruitment of leukocytes in a variety of inflammatory contexts (10). Together with our data showing high circulating levels of Mcpt4 during *P. y. yoelii* 17XNL infection (8), these observations suggested that Mcpt4 could contribute to early intestinal epithelial barrier damage and regulation of the observed type-2 immune response shift in this model. To test these hypotheses, we used *P. y. yoelii* 17XNL-infected *Mcpt4* ^-/-^ and *Mcpt4* ^+/+^ C57BL/6J mice as well as uninfected controls. Our data demonstrate for the first time that this human chymase ortholog orchestrates the regulation of malarial parasitemia and sexual stage parasite transmissibility as well as intestinal barrier function and host immunity to both infection and bacteremia.

## Materials and Methods

### Mouse Strains

Female, 7-8 week old *Mcpt4^-/-^* (13) and *Mcpt4* ^+/+^ C57BL/6J mice (Jackson Laboratory 000664) were housed in ventilated micro-isolator caging and provided food and water *ad libitum*. All procedures were approved by the Institutional Animal Care and Use Committee of the University of Idaho (IACUC Protocol 2020-10, approved 30 March 2020).

### Mouse Infection and Monitoring

*Mcpt4* ^-/-^ (n=44) and *Mcpt4^+/+^* (n=44) mice, distributed in three biological replicates, were injected intraperitoneally with 150 μL of *P. y. yoelii* 17XNL-infected red blood cells (iRBCs, 1 × 10^6^ parasites) as described (8). Control *Mcpt4* ^-/-^ (n=11) and *Mcpt4^+/+^* (n=10) mice were injected with equivalent numbers of uninfected RBCs. Infected mice were distributed into subgroups (n=11), in three replicates of 4, 4 and 3 mice, respectively, that were sacrificed at 4, 6, 8, or 10 days PI. Daily parasitemias were recorded from microscopic examination of Giemsa-stained thin blood films. Blood, plasma and ileum samples were collected and processed as described below.

### Bacterial 16S qPCR

Bacterial 16S copy numbers were determined by qPCR as described (8) for individual infected and uninfected mice from the three replicate studies (n=109). Briefly, whole blood was collected into EDTA on the day of necropsy and DNA was isolated using DNeasy Blood and Tissue kit (Qiagen) according to the manufacturer’s protocol. Samples were analyzed in triplicate using DNA SYBR Green/ROX qPCR Master Mix (2X) (Bio-Rad) with 16S primers and quantified against a 16S bacterial DNA plasmid standard curve using a QuantStudio 6 Flex Applied Biosystems qPCR as described (8).

### Intestinal Permeability

Intestinal permeability was determined as described (8) for individual mice in the third replicate (n=30), with 15 Mcpt4^-/-^ and 15 Mcpt4^+/+^ mice distributed in 5 subgroups each of 3 mice. Four subgroups were infected and sacrificed at 4, 6, 8 and 10 days PI and one subgroup served as uninfected controls. Briefly, fasted mice were orally gavaged with 4 kDa fluorescein isothiocyanate (FITC)-dextran and blood samples collected into EDTA at 3 h after gavage were analyzed using a microplate reader (Molecular Devices LLC, San Jose, CA) at excitation/emission wavelengths of 490/520 nm.

### Cytokines and Chemokines in Plasma and Ileum Samples

Plasma cytokine and chemokine concentrations (IL-1α, -1β, -2, -3, -4, -5, -6, -9, -10, -12p70, -12p40, -13, -17, IFN-γ, TNF-α, MCP-1, MIP-1α, MIP-1β, RANTES, eotaxin, GM-CSF, KC) were determined for individual infected and uninfected control mice in the three replicates (n=109) using a Bio-Plex Pro™ Luminex assay and analyzed on a Bio-Plex 200 system (Bio-Rad Laboratories) using Bio-Plex Manager software (Bio-Rad Laboratories) as described (8).

Ileum tissue sections (~2 cm long) collected at necropsy were weighed and processed for protein isolation using the Bio-Plex cell lysis kit (Bio-Rad, Hercules, CA). A lysing solution containing Bio-Plex cell lysis buffer and factors 1 and 2, along with a complete protease inhibitor cocktail (Millipore Sigma) was prepped and stored on ice until use. Tissue samples were kept on dry ice prior to lysing. Each tissue was rinsed thoroughly with 500 μL wash buffer provided in the lysis kit. The wash buffer was discarded and replaced with 500 μL of lysis solution. Ileum tissue was then lysed by sonication with a probe sonicator (Branson SFX150, Danbury, CT) using 8-12 pulses at 75 sec with 50% amplitude and 1 sec pause between each pulse. Once ileum tissue was homogenized, samples were transferred to dry ice for minimum of 10 min, then placed on ice to thaw slowly. After thawing, samples were sonicated in a water bath for 3 min then centrifuged at 4500 x g for 4 min at 4 °C. Supernatant was collected and placed on ice. Protein concentration was determined using Pierce BCA assay (ThermoFisher) based on the manufacturer’s protocol. Each sample was diluted 1:20 and absorbance was determined at 595 nm on an iMark Bio-Rad Microplate reader (Bio-Rad, Hercules, CA). Cytokine/chemokine profiles of the supernatants were determined by Bio-Plex Pro™ Luminex as for plasma samples and run in duplicate (14).

### ELISAs

Levels of plasma IgE (eBioscience; Thermo Fisher Scientific, Inc.), Mcpt1 (eBioscience) neutrophil elastase (NE) (Abcam) and myeloperoxidase (MPO) (eBioscience; Thermo Fisher Scientific, Inc.) were determined for individual mice in the three replicates (n=109) using commercial ELISAs according to manufacturer’s instructions and a microplate reader (Molecular Devices, LLC).

### Ileum Histochemistry

Ileum samples from individual mice in the first two replicates (n=79) were formalin-fixed and embedded in paraffin at the Washington Animal Disease Diagnostic Laboratory, Washington State University, and then subjected to detection of MC chymase activity by naphthol AS-D chloroacetate esterase (NASDCE) activity (Sigma-Aldrich) as described (8).

### Immunofluorescence of Mouse Ileum Sections

Paraffin-embedded ileum sections from three mice per time point (n=15) were heated for 20 min at 50 °C in a dry oven to soften the paraffin and then treated with xylene (three exchanges, 5 min each) to remove paraffin, followed by a wash/incubation sequence (100%, 90% and 70% ethanol, 5 min each) at room temperature to rehydrate the tissues, then the slides were rinsed in distilled water for 5 min. To enhance antigen retrieval, the slides were steamed for 40 min in Epitope Retrieval Solution (IHC WORLD). Slides were then washed with phosphate-buffered saline (PBS) and incubated for 1 h at room temperature in 5% bovine serum albumin (BSA) to block nonspecific binding sites. After three PBS washes, primary antibodies or isotype controls (1:100 rabbit anti-ZO-1, 1:100 monoclonal rat anti-E-cadherin, 1:100 rabbit primary isotype control, 1:100 mouse primary isotype control, eBioscience) were added, and the slides incubated overnight in a humidified chamber at 4°C. Following this incubation, slides were washed three time with PBS and fluorochrome-labeled secondary antibodies (1:150 chicken anti-rat Alexa Fluor 488 or 1:150 goat anti-rabbit Alexa Fluor 568; Invitrogen) were applied, and the slides were incubated for 1 h in a humidified chamber in the dark. After three PBS washes, coverslips were mounted with ProLong Gold antifade reagent (Invitrogen). Slides were viewed using a Nikon ECLIPSE Ti confocal microscope with a 40x objective. Quantitative analysis of immunofluorescence was performed by densitometry using NIH ImageJ software. A total of 10 to 15 high-power fields (HPF) per section were evaluated for each mouse from three independent biological replicates per time point.

### Mosquito Infection

*Anopheles stephensi* Liston were reared and allowed to feed on *P. y. yoelii* 17XNL-infected mice as described (15). Briefly, *Mcpt4* ^-/-^ (n=7) and *Mcpt4* ^+/+^ (n=7) mice from three biological replicates were infected as described above. At 3 days after mouse infection, levels of blood-stage parasitemia, gametocytemia and exflagellation were recorded and each mouse was used to infect 3-5 day old female *A. stephensi* (∼60 mosquitoes per mouse). At least 30 fed mosquitoes per mouse were dissected and microscopically examined for the presence of oocysts in the midgut at 10 days post-feeding. Midgut oocysts were counted by microscopic examination at 20X magnification.

### Statistical Analyses

Parasitemia and bacterial 16S DNA copies per µL of blood were analyzed by one-way ANOVA followed by Dunnett’s multiple comparisons test of each time point with the control group, or Šídák’s multiple comparisons test between *Mcpt4* ^-/-^ and *Mcpt4^+/+^* at 4, 6, 8 and 10 days PI. Plasma FITC-dextran, Mcpt1, IgE and NE, ileal MCs per HPF, plasma and ileal cytokines and chemokines and fluorescence intensity of E-cadherin and ZO-1 were analyzed with the Kruskal-Wallis test followed by Dunn’s multiple comparison of infected versus uninfected controls or *Mcpt4* ^-/-^ versus *Mcpt4^+/+^* mice at 4, 6, 8, and 10 days PI. Plasma MPO levels and percentages of infected mosquitoes were analyzed using Fisher’s exact test of *Mcpt4* ^-/-^ versus *Mcpt4^+/+^* mice. The numbers of oocysts per midgut from mosquitoes fed on *Mcpt4* ^-/-^ and *Mcpt4^+/+^* infected mice were compared using the Mann Whitney test. Parasitemia and gametocytemia at day 3 PI were analyzed using an unpaired t test of *Mcpt4* ^-/-^ versus *Mcpt4^+/+^* mice. P values ≤ 0.05 were considered significant. Correlations among parasitemia, blood 16S copies, plasma and ileal cytokines and chemokines as well as plasma IgE, Mcpt1, NE and MPO were assessed by Spearman test at 4, 6, 8, and 10 days PI. Network analysis was performed with Cytoscape^®^ software (www.cytoscape.org) version 3.8.2 using significant Spearman correlation coefficients (P ≤ 0.05) with parasitemia and 16S copies as main targets and MCs and immune factors as sources. Immune factors with a fold-change >1.5 relative to uninfected control were included in the network visualization analyses.

## Results

### Mcpt4 Deficiency Was Associated With Decreased *P. y. yoelii* 17XNL Parasitemia and Elevated Intestinal Permeability, But Unaltered Bacteremia Relative to *Mcpt4* ^+/+^ Mice

Previous studies showed that *Mcpt4* ^-/-^ mice exhibit increased basal intestinal barrier integrity relative to *Mcpt4* ^+/+^ mice (12), while *Mcpt4* ^-/-^ mice infected with the protozoan parasite *Giardia intestinali*s showed significantly reduced intestinal cytokine and alarmin expression and increased weight loss over time that was both more rapid and more severe relative to weight loss in infected *Mcpt4* ^+/+^ mice (16). Together with our data affirming the role of MCs in malaria-induced bacteremia (6, 7), we sought to determine whether Mcpt4 deficiency in the context of malaria was associated with alterations in parasitemia, intestinal permeability and infection-induced bacteremia over time.

**Figure 1:**
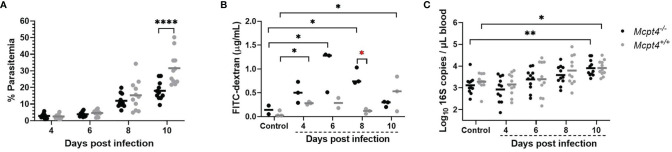
*Plasmodium y. yoelii* 17XNL parasitemia was decreased, intestinal permeability to FITC-dextran was increased, but bacteremia over time was not different in *Mcpt4* ^-/-^ relative to *Mcpt4* ^+/+^ mice. **(A)** Peripheral parasitemia following *P. y. yoelii* 17XNL infection, **(B)** intestinal permeability quantified as FITC-dextran in plasma of infected and uninfected mice after oral gavage and **(C)** bacterial 16S DNA copies/µL of blood in infected and uninfected mice. Each dot represents a single mouse. Parasitemia and bacterial 16S copies were determined for the three replicates (n=11 per group, per time point), while FITC-dextran was determined using the last replicate of mice only (n=3 mice per group per time point). Parasitemia and log 16S copies per μL of blood were analyzed using one-way ANOVA followed by Dunnett’s or Šídák’s multiple comparisons test; lines represent the mean. FITC-dextran data were analyzed with the Kruskal-Wallis test followed by Dunn’s test for multiple comparisons for each time point versus control or between *Mcpt4* ^-/-^ and *Mcpt4* ^+/+^ mice at specific time points; lines represent the median. P values ≤ 0.05 were considered significant. *P ≤ 0.05, **P ≤ 0.01, ****P ≤ 0.0001.

In our studies, *Mcpt4* ^-/-^ mice exhibited a trend towards reduced mean *P. y. yoelii* 17XNL parasitemia by 6 days PI (*Mcpt4* ^-/-^ mice = 3.9%, *Mcpt4* ^+/+^ mice = 4.6%) and 8 days PI (*Mcpt4* ^-/-^ mice = 12%, *Mcpt4* ^+/+^ mice = 15%) that was significantly lower relative to *Mcpt4* ^+/+^ mice by 10 days PI (*Mcpt4* ^-/-^ mice = 18%, *Mcpt4* ^+/+^ mice = 32%) (**Figure 1A**). Intestinal permeability, as measured by FITC-dextran translocation *in vivo* across the gut barrier (**Figure 1B**), was significantly increased in *Mcpt4* ^+/+^ mice relative to control at days 4 and 10 PI as previously observed (8). In *Mcpt4* ^-/-^ mice, FITC-dextran levels were significantly increased relative to control at 6 and 8 days PI, with a trend toward higher levels in *Mcpt4* ^-/-^ mice compared to *Mcpt4* ^+/+^ mice at 4 and 6 days PI that was significant at 8 days PI (**Figure 1B**). Levels in *Mcpt4* ^-/-^ mice decreased to control at 10 days PI (**Figure 1B**). Despite a pattern of increased intestinal permeability in *Mcpt4* ^-/-^ mice, infected *Mcpt4* ^-/-^ and *Mcpt4* ^+/+^ mice exhibited similarly increased bacterial 16S DNA levels in blood over time (**Figure 1C**). In contrast to Mcpt4-dependent host resistance to *G. intestinalis*-induced pathology, our data suggested that Mcpt4 suppresses the host immune response to *P. y. yoelii* 17XNL with alterations in parasitemia that overlapped with Mcpt4-dependent changes in intestinal permeability.

### *P. y. yoelii* 17XNL-Infected *Mcpt4* ^-/-^ Mice Exhibited Earlier Increases in Infection-Associated Ileal MCs and Higher Circulating Levels of IgE Relative to Infected *Mcpt4* ^+/+^ Mice

Together with data showing that Mcpt4 controls intestinal barrier function (12) and the potential for autocrine regulation of MCs by chymase/Mcpt4 (17), our observations of MC-dependent malaria-induced disruption of the intestinal barrier suggested that Mcpt4 might mediate intestinal MC accumulation and activation during *P. y. yoelii* 17XNL infection. To test this hypothesis, we counted NASDCE-positive ileal MC and quantified plasma Mcpt1 as an additional marker of MC activation in control uninfected and *P. y. yoelii* 17XNL-infected *Mcpt4* ^-/-^ and *Mcpt4* ^+/+^ mice.

**Figure 2:**
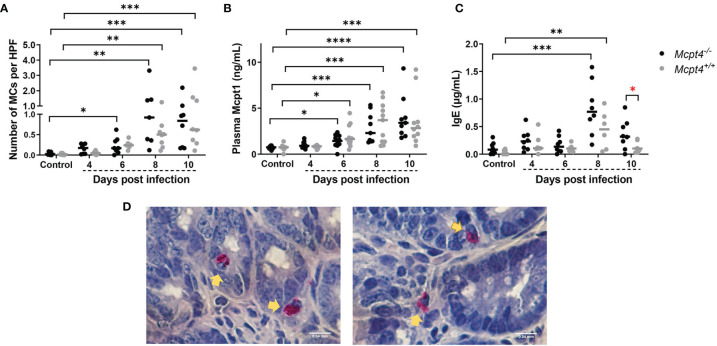
*Plasmodium y. yoelii* 17XNL-infected *Mcpt4^-/-^* mice exhibited earlier infection-associated increases in ileal MCs and higher circulating levels of IgE relative to infected *Mcpt4* ^+/+^ mice. **(A)** Numbers of ileal MCs per high-powered field (HPF) from naphthol AS-D chloroacetate esterase (NASDCE) stained sections from uninfected control mice and infected *Mcpt4^-/-^* and *Mcpt4* ^+/+^ mice. **(B)** Plasma MC protease 1 (Mcpt1) concentrations as determined by ELISA. **(C)** Plasma IgE concentrations as determined by ELISA. **(D)** Representative stained MCs (pink cells indicated by yellow arrows) in ileum of *Mcpt4^-/-^* (left) and *Mcpt4* ^+/+^ (right) mice at 8 days PI. Number of MCs and IgE levels in plasma were determined for biological replicates 1 and 2 (n=8 mice per group per time point), while plasma Mcpt1 levels were determined for the three replicates (n=11 mice per group per time point). Data were analyzed with the Kruskal-Wallis test followed by Dunn’s multiple comparison for each time point versus control or between *Mcpt4^-/-^* and *Mcpt4* ^+/+^ mice at specific time points; lines represent the median. P values ≤ 0.05 were considered significant. *P ≤ 0.05, **P ≤ 0.01; ***P ≤ 0.001, ****P ≤ 0.0001.

An infection-associated increase in ileal MCs was observed in *Mcpt4* ^-/-^ mice at 6 days PI relative to uninfected controls, while no increase was noted in *Mcpt4* ^+/+^ mice at this time point (**Figures 2A, D**). By 8 and 10 days PI, both infected *Mcpt4* ^+/+^ and *Mcpt4* ^-/-^ mice exhibited significantly increased ileal MCs relative to uninfected controls (**Figures 2A, D** and **Figure 1S**). While Mcpt1 levels rose over time with infection, there were no differences in plasma Mcpt1 levels between *Mcpt4* ^+/+^ and *Mcpt4^-/-^* mice at any time point PI (**Figures 2A, B**). Elevated levels of IgE were observed at 8 days PI in both *Mcpt4* ^+/+^ and *Mcpt4* ^-/-^ mice relative to control uninfected mice with a trend towards higher levels in *Mcpt4* ^-/-^ mice (**Figure 2C**). By 10 days PI, IgE levels dropped in both groups and were no longer significant relative to control uninfected mice, but IgE levels in infected *Mcpt4* ^-/-^ mice were significantly higher than levels in infected *Mcpt4* ^+/+^ mice (**Figure 2C**). Mcpt4 deficiency has been associated with increased serum IgE levels in a model of airway allergic inflammation (18), suggesting an effect of Mcpt4 that is conserved in our model. Overall, our data indicate that significantly increased intestinal MCs appeared earlier in infected *Mcpt4* ^-/-^ mice. Further, contrary to our hypothesis, MC activation is not disabled by *Mcpt4* depletion, a state which is also associated with a stronger and more sustained IgE response later in infection.

### Mcpt4 Deficiency Was Associated With Altered Patterns of Ileal Adherens Junctions During *P. y. yoelii* 17XNL Infection Relative to *Mcpt4* ^+/+^ Mice

**Figure 3:**
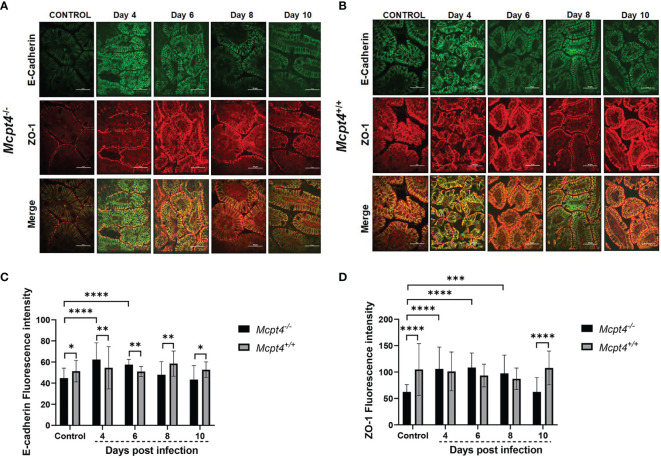
*Mcpt4^-/-^* mice exhibited altered patterns of ileal adherens junctions during *P. y. yoelii* 17XNL infection relative to *Mcpt4* ^+/+^ mice. Representative images from *P. y. yoelii* 17XNL-infected *Mcpt4^-/-^* **(A)** and *Mcpt4* ^+/+^ **(B)** mice and uninfected controls. The top, middle and bottom rows contain images of E-cadherin (green) and ZO-1 (red) staining and merged images. Scale bars represent 50 μm length. Quantitation of E-cadherin **(C)** and ZO-1 **(D)**. Quantitative analysis of fluorescence was performed using NIH ImageJ software. Data are represented as mean ± SD from 10-15 HPF per mouse from three independent biological replicates (n=5 *Mcpt4^-/-^* mice and n=5 *Mcpt4* ^+/+^ mice per replicate). Data were analyzed with the Kruskal-Wallis test followed by Dunn’s multiple comparison for each time point versus control or between *Mcpt4^-/-^* and *Mcpt4* ^+/+^ mice at specific time points. P values ≤ 0.05 were considered significant. *P ≤ 0.05, **P ≤ 0.01, ***P ≤ 0.001, ****P ≤ 0.0001.

Differences in intestinal permeability can be positively associated with physical structure of the intestinal epithelial barrier and MCs are important regulators of the integrity of this barrier (19, 20). Accordingly, we evaluated distribution and intensity of zonula occludens 1 (ZO-1) and E-cadherin in the ileal epithelium as indicators of the integrity of intracellular tight junctions (TJs) and adherens junctions (AJs), respectively, in tissue sections from uninfected and infected *Mcpt4* ^-/-^ and *Mcpt4* ^+/+^ mice (**Figures 3A, B**).

In uninfected control mice, E-cadherin and ZO-1 staining intensities were significantly lower in *Mcpt4* ^-/-^ relative to *Mcpt4* ^+/+^ mice (**Figures 3C, D**). E-cadherin staining was significantly increased by parasite infection in *Mcpt4* ^-/-^ mice at 4 and 6 days PI and these increases were significant relative to infected *Mcpt4* ^+/+^ mice within these time points (**Figure 3C**). E-cadherin staining in infected *Mcpt4* ^-/-^ mice was significantly lower than in infected *Mcpt4* ^+/+^ mice at days 8 and 10 PI, but staining intensities at these time points were not different from uninfected controls. ZO-1 staining was significantly increased in *Mcpt4* ^-/-^ mice at 4, 6, and 8 days PI relative to uninfected controls, but staining intensity was not different from infected *Mcpt4* ^+/+^ mice within these time points (**Figure 3D**). ZO-1 staining in *Mcpt4* ^-/-^ mice was reduced relative to infected *Mcpt4* ^+/+^ mice at day 10 PI, but staining intensity at this time point was not different from uninfected controls. In looking more closely at the patterns of staining in infected *Mcpt4* ^-/-^ versus infected *Mcpt4* ^+/+^ mice, intracellular E-cadherin staining was more evident in infected *Mcpt4^-/-^* versus infected *Mcpt4* ^+/+^ mice at 4 and 6 days PI, while ZO-1 staining remained localized to the cell surface (merge, **Figures 3A, B** and **Figure 2S**). Further, intracellular E-cadherin staining at days 4 and 6 PI in *Mcpt4^-/-^* mice corresponded with trends toward increased permeability to FITC-dextran relative to *Mcpt4* ^+/+^ at days 4 and 6 PI that were followed by a significant infection-associated increase at 8 days PI (**Figure 1B**). In a mouse model of irritable bowel disease (IBD; *C1orf106* -/-), intestinal epithelial cells from mutant mice showed increased intracellular versus cell surface staining of E-cadherin in the absence of significant changes in the distribution of ZO-1 (21). Although this pattern was not associated with increased permeability to FITC-dextran *in vivo*, the *C1orf106* -/- intestinal epithelium was significantly more permeable *in vitro* to the smaller compound lucifer yellow and *C1orf106* -/- mice exhibited significantly increased extraintestinal translocation of the enteric pathogen *Citrobacter rodentium* relative to *C1orf106* +/+ mice (21). Taken together with these observations, the shift in intensity and localization of ileal E-cadherin staining in infected *Mcpt4^-/-^* mice relative to infected *Mcpt4* ^+/+^ mice provide an interesting foundation for future studies.

### Mcpt4 Deficiency Was Associated With Decreased Neutrophil Activation During *P. y. yoelii* 17XNL Infection

**Figure 4:**
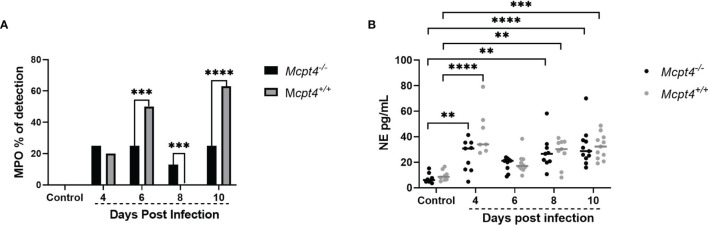
*Mcpt4^-/-^* mice exhibited significantly decreased neutrophil activation at 6 and 10 days following *P. y. yoelii* 17XNL infection. **(A)** Plasma myeloperoxidase (MPO) detection by ELISA, represented as percentage of positive plasma samples at 4, 6, 8, and 10 days PI. **(B)** Plasma neutrophil elastase (NE) as determined by ELISA. MPO and NE were determined for the three biological replicates (n=11 mice per group per time point). MPO data were analyzed with Fisher’s exact test and NE data were analyzed with the Kruskal-Wallis test followed by Dunn’s multiple comparison for each time point versus control or between *Mcpt4^-/-^* and *Mcpt4* ^+/+^ mice at specific time points. P values ≤ 0.05 were considered significant. **P ≤ 0.01; ***P ≤ 0.001, ****P ≤ 0.0001.

The association of altered ileal adherens junctions in a mouse model of IBD with both increased intestinal permeability and increased extraintestinal translocation of gut bacteria (21) pointed to a clear difference compared with our data. That is, relative to *Mcpt4* ^+/+^ mice, parasitemia was reduced in *Mcpt4* ^-/-^ mice (**Figure 1A**), adherens junctions were altered (**Figures 3A**–**C**) and intestinal permeability to FITC-dextran was markedly increased (**Figure 1B**), but there were no differences in blood 16S copies over time between infected *Mcpt4^-/-^* and *Mcpt4* ^+/+^ mice (**Figure 1C**). Human chymase and mouse Mcpt4 can induce the inflammatory recruitment and accumulation of neutrophils (10), cells which could provide protection against infection but also contribute to disease pathology (22). To examine neutrophil activation in the context of parasite infection and Mcpt4 deficiency, we examined levels of plasma myeloperoxidase (MPO) and neutrophil elastase (NE) (23), antimicrobial effectors frequently used as markers for neutrophil activation (24, 25). The proportions of MPO-positive mice varied across infection with no difference at 4 days PI, decreased MPO detection in *Mcpt4^-/-^* versus *Mcpt4* ^+/+^ mice at 6 days PI, low MPO detection in *Mcpt4^-/-^* mice relative to no detection in *Mcpt4* ^+/+^ mice at 8 days PI and decreased MPO detection in *Mcpt4^-/-^* versus *Mcpt4* ^+/+^ mice at 10 days PI (**Figure 4A**). Levels of plasma NE were not different between *Mcpt4* ^-/-^ and *Mcpt4* ^+/+^ mice, with elevated levels in both groups at 4, 8, and 10 days PI relative to uninfected controls (**Figure 4B**). While robust MPO detection at 6 and 10 days PI in infected *Mcpt4* ^+/+^ mice relative to *Mcpt4^-/-^* mice affirmed a role for Mcpt4 in neutrophil activation, these observations were not consistent with increased parasitemia in *Mcpt4* ^+/+^ mice relative to the *Mcpt4^-/-^* mice at 10 days PI (**Figure 1A**) and with the lack of differences in blood 16S copies in *Mcpt4* ^-/-^ and *Mcpt4* ^+/+^ mice over time (**Figure 1C**).

### Cytokine Profiles During *P. y. yoelii* 17XNL Infection Suggested an Earlier Type-1 Immune Response in *Mcpt4* ^-/-^ Mice and a Type-2 Response in *Mcpt4* ^+/+^ Mice

Given that Mcpt4 can degrade a variety of pro-inflammatory cytokines (10), we hypothesized that a deficiency in this protease could be correlated with increased levels of cytokines associated with host protection against parasite infection and bacteremia. To test this hypothesis, we examined cytokine and chemokine levels in both plasma and ileum tissue over time to reveal patterns of systemic and local responses, respectively, following *P. y. yoelii* 17XNL infection in *Mcpt4* ^-/-^ and *Mcpt4* ^+/+^ mice.

**Figure 5:**
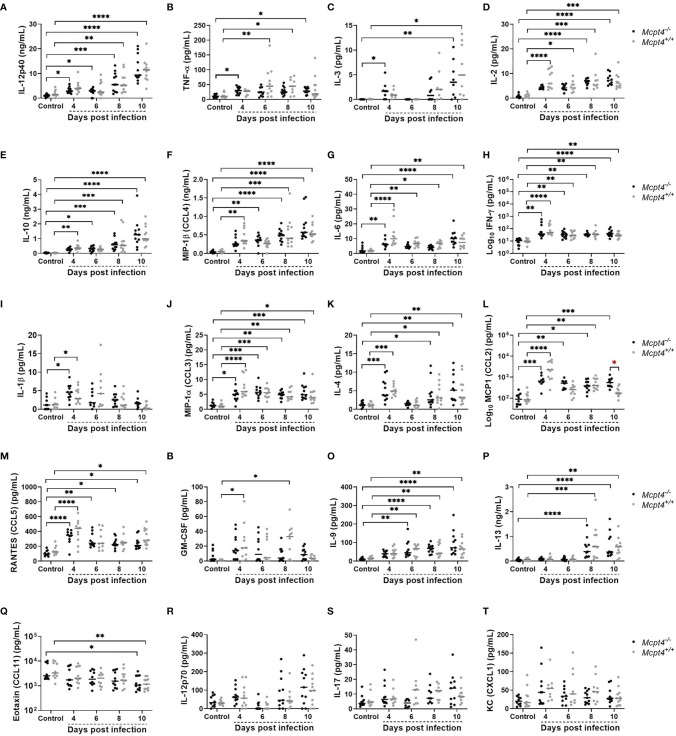
Plasma cytokines and chemokines in *P. y. yoelii* 17XNL-infected *Mcpt4^-/-^* and *Mcpt4* ^+/+^ mice and uninfected controls. **(A–T)** The x axis represents the time points in days after infection, and the y axis represents the concentrations of IL-12p40 **(A)**, TNF-α **(B)**, IL-3 **(C)**, IL-2 **(D)**, IL-10 **(E)**, MIP1β (CCL4) **(F)**, IL-6 **(G)**, IFN-γ **(H)**, IL-1β **(I)**, MIP1α (CCL3) **(J)**, IL-4 **(K)**, MCP-1 (CCL2) **(L)**, RANTES (CCL5) **(M)**, GM-CSF **(N)**, IL-9 **(O)** IL-13 **(P)** Eotaxin (CCL11) **(Q)**, IL-12p70 **(R)**, IL-17 **(S)** and KC (CXCL1) **(T)**. Each dot represents a single mouse. Plasma cytokines were determined for the three biological replicates (n=11 mice per group per time point). Data were analyzed with the Kruskal-Wallis test followed by Dunn’s multiple comparison for each time point versus control or between *Mcpt4^-/-^* and *Mcpt4* ^+/+^ mice at specific time points. P values ≤ 0.05 were considered significant. *P ≤ 0.05, **P ≤ 0.01; ***P ≤ 0.001, **** P ≤ 0.0001.

The pro-inflammatory cytokines IL-12p40 (**Figure 5A**) and TNF-α (**Figure 5B**) as well as the MC growth factor IL-3 (**Figure 5C**) were significantly increased in plasma of *Mcpt4* ^-/-^ mice compared to uninfected controls early after infection (4 days PI). In *Mcpt4* ^+/+^ mice, IL-2 (**Figure 5D**), IL-10 (**Figure 5E**) and MIP-1β/CCL4 (**Figure 5F**) were significantly increased compared to uninfected controls by the same days PI as observed previously (8). Pro-inflammatory IL-6 (**Figure 5G**), IFN-γ (**Figure 5H**) and IL-1β (**Figure 5I**), the type 1 chemokine MIP-1α/CCL3 (**Figure 5J**), the type 2 regulatory cytokine IL-4 (**Figure 5K**), MCP-1/CCL2 (**Figure 5L**), RANTES/CCL5 (**Figure 5M**) and the granulocytic growth factor GM-CSF (**Figure 5N**) were increased in plasma at 4 days PI in both *Mcpt4* ^-/-^ and *Mcpt4* ^+/+^ mice. Within time points, MCP-1/CCL2 was significantly different between *Mcpt4* ^-/-^ and *Mcpt4* ^+/+^ mice at 10 days PI (**Figure 5L**). Increased plasma levels of IL-9 (**Figure 5O**) and IL-13 (**Figure 5P**), both of which are associated with type 2 immunity and MC activation, were observed at 6 and 8 days PI, respectively, in both *Mcpt4* ^-/-^ and *Mcpt4* ^+/+^ mice. Eotaxin/CCL11 was significantly decreased relative to control by 10 days PI in both *Mcpt4* ^-/-^ and *Mcpt4* ^+/+^ mice (**Figure 5Q**). No differences relative to control were observed for IL-12p70 (**Figure 5R**), IL-17 (**Figure 5S**) and KC (CXCL1) (**Figure 5T**). Overall, these results suggested an early type 1 response in infected *Mcpt4* ^-/-^ mice and skewing towards a type-2 response in infected *Mcpt4* ^+/+^ mice over the same time period. Based on MCP-1/CCL2 regulation of the migration and infiltration of monocytes and macrophages (26), an early type-1 response and elevated MCP-1/CCL2 in *Mcpt4* ^-/-^ relative to *Mcpt4* ^+/+^ mice could explain the significant reduction in parasitemia in *Mcpt4^-/-^* mice.

**Figure 6:**
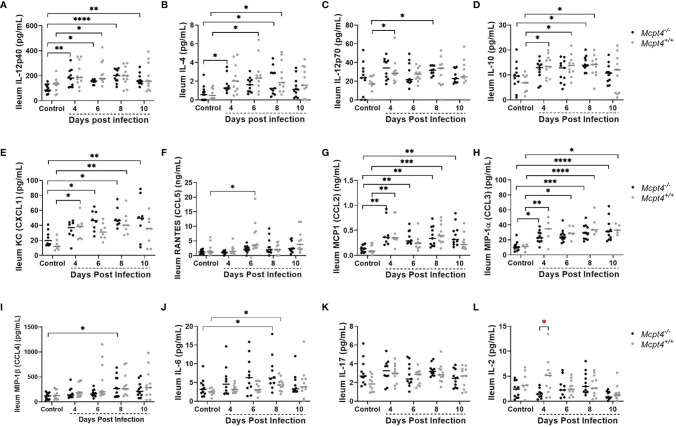
Ileal cytokines and chemokines in *P. y. yoelii* 17XNL-infected *Mcpt4^-/-^* and *Mcpt4* ^+/+^ mice and uninfected controls. **(A–L)** The x axis represents the time points in days after infection, and the y axis represents the concentrations of IL-12p40 **(A)**, IL-4 **(B)**, IL-12p40 **(C)**, IL-10 **(D)**, KC (CXCL1) **(E)**, RANTES (CCL5) **(F)**, MCP-1 (CCL2) **(G)**, MIP1α (CCL3) **(H)**, MIP1β (CCL4) **(I)**, IL-6 **(J)**, IL-17 **(K)** and IL-2 **(L)**. Each dot represents a single mouse. Cytokines levels in ileum were determined for the three biological replicates (n=11 mice per group per time point). Data were analyzed with the Kruskal-Wallis test followed by Dunn’s multiple comparison for each time point versus control or between *Mcpt4^-/-^* and *Mcpt4* ^+/+^ mice at specific time points. P values ≤ 0.05 were considered significant. *P ≤ 0.05, **P ≤ 0.01, ***P ≤ 0.001, ****P ≤ 0.0001.

In the ileum, early expression (4 days PI) of IL-12p40 (**Figure 6A**) and IL-4 (**Figure 6B**) were observed in *Mcpt4* ^-/-^ mice, while increased expression of IL-12p70 (**Figure 6C**) (antagonist of IL-12p40), IL-10 (**Figure 6D**) and the neutrophil chemoattractant KC/CXCL1 (**Figure 6E**) were observed in *Mcpt4* ^+/+^ mice at the same time point, followed by RANTES/CCL5 (**Figure 6F**) at 6 days PI in the same group of animals. Early (4 days PI) increases in MCP-1/CCL2 (**Figure 6G**) and MIP-1α/CCL3 (**Figure 6H**) were observed in both *Mcpt4^-/-^* and *Mcpt4* ^+/+^ mice, followed by later (8 days PI) increases in MIP-1β/CCL4 (**Figure 6I**) in *Mcpt4* ^-/-^ mice and IL-6 (**Figure 6J**) in both *Mcpt4* ^-/-^ and *Mcpt4* ^+/+^ mice. No differences relative to control were observed for IL-17 (**Figure 6K**) and IL-2 (**Figure 6L**). In general, ileum cytokine levels were lower than plasma levels, but as observed in plasma samples, the local ileal immune response in *Mcpt4* ^-/-^ mice was more consistent with a type-1 immune response (IL-12p40), while the local response in *Mcpt4* ^+/+^ mice was more type-2 (IL-12p70, IL-10).

### Correlation Analyses and Network Visualization Affirmed a Stronger Earlier Immune Response in *Mcpt4* ^-/-^ Mice Relative to *Mcpt4* ^+/+^ Mice

**Figure 7:**
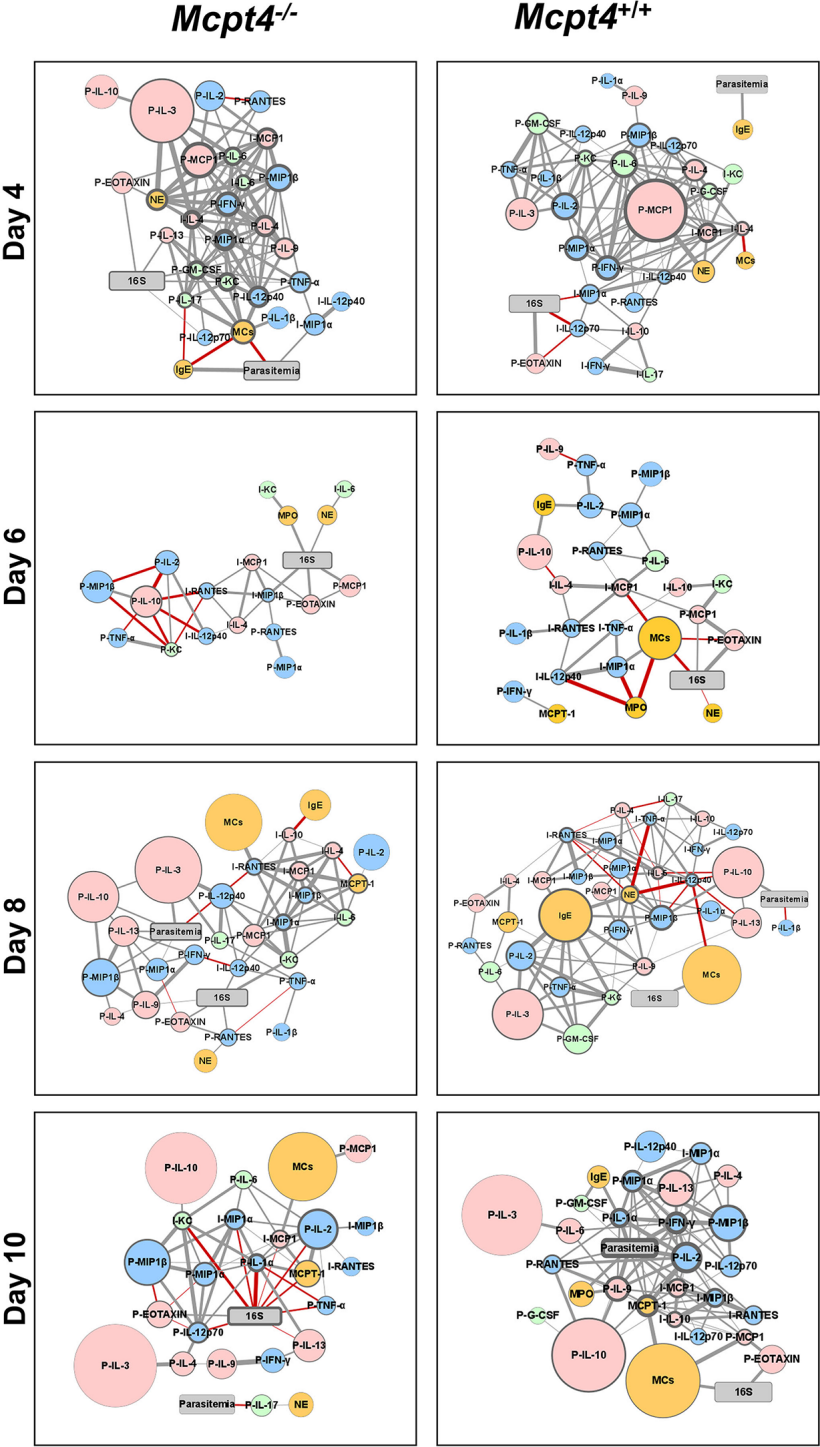
Network visualization of *Mcpt4^-/-^* and *Mcpt4* ^+/+^ mouse phenotypes during *P. y. yoelii* 17XNL infection. Network visualization of significant correlations (**Figure S3**) at different times post-infection in *Mcpt4^-/-^* mice (left) and *Mcpt4* ^+/+^ mice (right). The size of a circular node represents relative fold change (larger circle = larger fold change) of a parameter calculated as average levels in infected mice/average levels in control uninfected mice. The border width represents the degree or number of connections (the higher the number of connected edges, the thicker the edge). Parasitemia and 16S nodes are presented in gray, blue nodes are pro-inflammatory cytokines and chemokines (type-1 immune response), pink nodes are anti-inflammatory or regulatory cytokines and chemokines (type-2 immune response), green nodes are type-17 immunity-related cytokines and chemokines, orange nodes represent cells and cells markers (MCs, Mcpt1, MPO and NE). Gray strokes connect nodes with positive correlations and red strokes reflect negative correlations. Increasing stroke line width reflects increasing Spearman’s correlation value.

To visualize the networked host response, we first constructed correlation matrices for *Mcpt4* ^-/-^ and *Mcpt4* ^+/+^ mice to identify significant positive and negative relationships over time between parasitemia and blood 16S copies with ileal MC numbers, levels of plasma and ileal cytokines and chemokines, plasma IgE, plasma Mcpt1, MPO and NE (**Figure S3**). Significant correlations (P ≤ 0.05) with fold changes > 1.5 relative to uninfected controls were used to build interaction networks by day to better understand these complex host responses (**Figure 7**).

At 4 days PI, *Mcpt4* ^-/-^ mice exhibited a more balanced type-1/type-2 immune response relative to *Mcpt4* ^+/+^ mice as evidenced by the presence of IFN-γ at the center of the host response network and the presence of plasma IL-17. In *Mcpt4* ^-/-^ mice, MCs were also networked directly and negatively correlated with parasitemia, suggesting early MC regulation of parasitemia. In *Mcpt4* ^+/+^ mice, no correlations between parasitemia and any cytokines or chemokines were observed at 4 days PI, and the host response appears to be driven primarily by blood 16S copies at the level of the ileum. Plasma IgE was positively correlated with parasitemia in both *Mcpt4^-/-^* and *Mcpt4* ^+/+^ mice, with this being the only direct correlation with parasitemia in *Mcpt4* ^+/+^ mice.

By 6 days PI, no correlations were observed between parasitemia and any cytokines or chemokines in *Mcpt4^-/-^* or *Mcpt4* ^+/+^ mice. The host responses in both *Mcpt4^-/-^* and *Mcpt4* ^+/+^ mice were 16S-dependent with blood 16S copies directly and positively correlated with plasma MCP-1/CCL2 and eotaxin/CCL11 and ileal MIP-1β/CCL4 as well as NE and MPO in *Mcpt4^-/-^* mice. In *Mcpt4* ^+/+^ mice, blood 16S copies were negatively correlated with NE and MCs and positively correlated with plasma eotaxin/CCL11 and MCP-1/CCL2.

By 8 days PI, parasitemia in *Mcpt4^-/-^* was correlated with larger fold changes in plasma type-2 cytokines (IL-3, IL-13 and IL-10) and chemokines (MIP-1α/CCL3 and MIP-1β/CCL4), while blood 16S copies were correlated directly and positively with plasma eotaxin/CCL11, IL-9, MCP-1/CCL2 and RANTES/CCL5 and ileal KC/CXCL1. Plasma MCP-1/CCL2 and ileal KC/CXCL1 connected blood 16S copies with a series of ileal cytokines and chemokines (IL-6, IL-10, MCP-1/CCL2, MIP-1α/CCL3, MIP-1β/CCL4 and RANTES/CCL5) and with MCs. In *Mcpt4* ^+/+^ mice, parasitemia was positively correlated with plasma IL-10 and negatively with plasma IL-1β while blood 16S copies were positively correlated with MCs and Mcpt1

By 10 days PI, parasitemia in *Mcpt4^-/-^* mice was correlated only with the pro-inflammatory cytokine IL-17 in the plasma and this correlation was negative. The overall host response in *Mcpt4^-/-^* mice appears to be driven by blood 16S copies, with a notable number of direct negative correlations with plasma and ileal cytokines and chemokines. In *Mcpt4* ^+/+^ mice, parasitemia is the overwhelming focal point of the host immune response, with correlations between type 1 (IFN-γ) and type 2 (IL-10 and IL-13) cytokines and chemokines as well as MPO. In *Mcpt4* ^+/+^ mice, MCs connect with parasitemia through Mcpt1 and are positively correlated with blood 16S copies.

### Mcpt4 Deficiency Was Associated With Increased *P. y. yoelii* 17XNL Transmission to *Anopheles stephensi*

**Figure 8:**
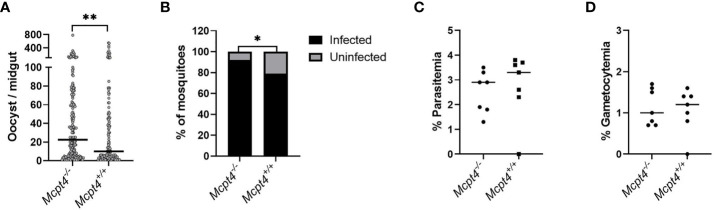
Transmission of *P. y. yoelii* 17XNL from infected *Mcpt4^-/-^* and *Mcpt4* ^+/+^ mice to *Anopheles stephensi*. Panels **(A, B)**, respectively, represent the numbers of oocysts per midgut and proportions of infected mosquitoes in three independent biological replicates. Panels **(C, D)**, respectively, illustrate the percentages of mouse erythrocytes infected with asexual stage *P. y. yoelii* 17XNL and sexual stage gametocytes on the day of mosquito infection (day 3 PI). Parasite transmission was determined in three biological replicates (n=7 mice per group, 30 fed mosquitoes per mouse). The numbers of midgut oocysts in mosquitoes fed on *Mcpt4^-/-^* and *Mcpt4* ^+/+^ infected mice were analyzed by Mann Whitney test. The percentages of infected mosquitoes (mosquitoes with zero oocysts excluded) were analyzed with Fisher’s exact test. Parasitemia and gametocytemia data were analyzed using unpaired t tests. P values ≤ 0.05 were considered significant. *P ≤ 0.05, **P ≤ 0.01.

The interruption of malaria parasite transmission from infected humans to mosquitoes is one of the main challenges for malaria control and elimination programs. It has been established that naturally acquired immune responses to gametocytes can affect parasite infectiousness to *Anopheles* mosquitoes (27). To identify the effects of Mcpt4 on gametocytemia and parasite transmission in our model, we exposed infected *Mcpt4^-/-^* and *Mcpt4* ^+/+^ mice to 3-5 day old female *A. stephensi*. In three biological replicates, midgut oocyst numbers were modestly, but significantly higher in *A. stephensi* fed on *Mcpt4^-/-^* mice relative to mosquitoes fed on infected *Mcpt4* ^+/+^ mice (**Figure 8A**). Similarly, a higher percentage of mosquitoes were infected following feeding on *Mcpt4^-/-^* versus *Mcpt4* ^+/+^ mice (**Figure 8B**). Surprisingly, there were no differences in exflagellation (not shown), parasitemia (**Figure 8C**) or gametocytemia (**Figure 8D**) between infected *Mcpt4^-/-^* and *Mcpt4* ^+/+^ mice on the day of mosquito infection. Taken together, these results suggest that the mouse chymase Mcpt4 alters parasite transmissibility without discernable effects on peripheral gametocytemia.

## Discussion

In a previous study, we observed that circulating levels of mouse Mcpt4 were significantly increased at 4 days (10-fold above control) and 8 days (6-fold above control) after *P. y. yoelii* 17XNL infection (8) with sharply increased intestinal MCs relative to control on the same days. Mcpt4 has been associated with MC-dependent regulation and homeostasis of the intestinal epithelium (12), limiting inflammation in a sepsis model (28), modulating scar development (29, 30) and regulation of the intestinal inflammatory response to *G. intestinali*s (16), but the role of Mcpt4 in malaria has not been studied. Here we provide data that suggest for the first time that Mcpt4, the ortholog of human chymase, promotes a type-2 immune response, regulates alterations to the intestinal barrier associated with the development of parasitemia and bacteremia and restricts parasite transmission to *A. stephensi*.

In general, our network analyses revealed patterns and complexity over time that were consistent with an interpretation of temporally accelerated control of parasitemia and a stronger systemic response to bacteremia in *Mcpt4^-/-^* mice relative to *Mcpt4* ^+/+^ mice. The *Mcpt4^-/-^* interaction network had a substantially larger number of nodes around parasitemia at days 4 and 8 PI and around blood 16S copies at 4, 8, and 10 days PI than observed in *Mcpt4* ^+/+^ mice. At 4 days PI, the *Mcpt4* ^+/+^ network had a single node connected with parasitemia; a strong network focus on parasitemia was delayed in *Mcpt4* ^+/+^ mice until 10 days PI. In contrast, blood 16S copies were the major network focus in *Mcpt4^-/-^* mice at 10 days PI, while parasitemia was separated from the primary network with only a few interacting nodes, a pattern consistent with significantly reduced parasitemia in *Mcpt4^-/-^* mice at this time (**Figure 1A**). Intriguingly, the networks for both *Mcpt4^-/-^* mice and *Mcpt4* ^+/+^ mice at 6 days PI were relatively small and lacked nodes correlated with parasitemia. We previously inferred that this time point is associated with a shift between innate and adaptive immunity in our infection model (8), which appears to be the case herein given the significant increase in IgE for both *Mcpt4^-/-^* and *Mcpt4* ^+/+^ mice at 8 days PI (**Figure 2C**). However, the nearly identical levels of parasitemia preceding (4 days) and coinciding with this shift (6 days; **Figure 1A**) suggested that another insult was responsible for the divergence of the *Mcpt4^-/-^* and *Mcpt4* ^+/+^ networks over time. Based on our data, we suggest that this insult was increased intestinal permeability in *Mcpt4^-/-^* mice that trended higher than *Mcpt4* ^+/+^ mice at 4 and 6 days PI, a pattern that was significantly increased by 8 days PI (**Figure 1B**). We infer from our network analyses that this barrier disruption likely contributed to an earlier and broader systemic immune response in the *Mcpt4^-/-^* mice relative to the *Mcpt4* ^+/+^ mice that maintained blood 16S copies at levels that were not different with *Mcpt4* ^+/+^ levels at any time point and that significantly reduced parasitemia by 10 days PI in *Mcpt4^-/-^* mice. We discuss these patterns and complexity in more detail below to support our inferences.

In contrast to previous studies that showed that *Mcpt4^-/-^* mice exhibited increased basal intestinal barrier integrity relative to *Mcpt4* ^+/+^ mice (12), we observed an infection-associated decrease in intestinal barrier integrity in *Mcpt4^-/-^* mice (**Figure 1B**) and altered ileal E-cadherin staining (**Figures 3C, D**) that was similar to a pattern associated with increased enteric bacterial translocation in another mouse model (21). Ileal MC numbers in *Mcpt4^-/-^* mice were significantly increased relative to control at 6 days PI, two days earlier than observed in *Mcpt4* ^+/+^ mice, but both *Mcpt4^-/-^* and *Mcpt4* ^+/+^ mice exhibited significantly increased ileal MCs relative to control at 8 and 10 days PI (**Figure 2A**). The trend toward increased IgE levels in *Mcpt4^-/-^* mice relative to *Mcpt4* ^+/+^ at 8 days PI became significant by 10 days PI (**Figure 2C**) and could predict increasingly greater MC activation in *Mcpt4^-/-^* versus *Mcpt4* ^+/+^ mice. Intestinal permeability in *Mcpt4^-/-^* mice returned to baseline by 10 days PI, however, with a trend towards reduced permeability relative to *Mcpt4* ^+/+^ mice at this time point (**Figure 1C**), suggesting a homeostatic correction in *Mcpt4^-/-^* mice later in infection. The role of MCs as key modulators of barrier function and homeostasis of the intestinal mucosa has been previously established (20) and we can now add that a leaky gut in *Mcpt4^-/-^* mice is associated with substantial changes in the host immune response to malaria parasite infection.

The observed reduction in parasitemia in *Mcpt4^-/-^* mice was associated with an earlier type 1 immune response relative to *Mcpt4* ^+/+^ mice, with increased plasma IL-12p40 (**Figure 5A**) and TNF-α (**Figure 5B**) by 4 days PI. Both of these cytokines, as well as IFN-γ, were indirectly correlated with parasitemia through MCs, GM-CSF, KC/CXCL1 and IL-17 at the same time point (**Figure 7**). In contrast to *Mcpt4^-/-^* mice, plasma IL-10 in *Mcpt4* ^+/+^ mice was significantly increased relative to controls at 4 days PI, two days earlier than in *Mcpt4^-/-^* mice (**Figure 5E**), suggesting earlier immune down-regulation in *Mcpt4* ^+/+^ mice. By 8 days PI, parasitemia in *Mcpt4^-/-^* mice was correlated mainly with type-2 cytokines including large fold-changes in IL-3 and IL-10, which were also notable in *Mcpt4* ^+/+^ mice. However, the *Mcpt4^-/-^* network also included indirect correlations with the chemokines MIP-1α/CCL3 and MIP-1β/CCL4, as well as persistence of IL-12p40, IFN-γ and TNF-α (**Figure 7**). Pro-inflammatory cytokines, particularly IL-12 and INF-γ, have been associated with protection and parasite clearance in malaria (31, 32). While the role of IL-17 in parasite clearance is not clear, MCs are reported to be an important source of this cytokine in addition to Th17 cells (33–35) and IL-17 levels has been associated with protection against *Plasmodium berghei* (ANKA strain) (36) and severe disease in falciparum malaria (37). Moreover, tryptase co-released from stimulated human MCs following FcϵRI cross-linking has been associated with *in vitro* degradation of MCP-1/CCL2 (38), an important chemoattractant of monocytes and macrophages (26) that could also participate in parasite clearance. Some studies have suggested that Mcpt4 also cleaves MCP-1 (39), but others have not shown this (40–42), leaving this biology unclear. IgE levels trended higher at 8 days PI in *Mcpt4^-/-^* mice and were significantly higher than *Mcpt4* ^+/+^ levels at 10 days PI (**Figure 2C**), suggesting an association between IgE, activated MCs and elevated MCP-1/CCL2 in *Mcpt4^-/-^* mice. Together, our data suggest that early MC synthesis of Mcpt4 suppresses the host immune response to *P. y. yoelii* 17XNL infection, perhaps *via* degradation of pro-inflammatory TNF-α, IFN-γ and MCP-1/CCL2 (28, 39) and promotion of a type-2 immune response.

Inflammatory cytokines and chemokines can enhance neutrophil responsiveness to circulating bacteria, but in the context of malaria, this response can be compromised (43), so we monitored circulating levels of MPO and NE as markers of neutrophil activation in *Mcpt4^-/-^* and *Mcpt4* ^+/+^ mice over the course of *P. y. yoelii* 17XNL infection. Surprisingly, MPO detection was reduced in *Mcpt4^-/-^* versus *Mcpt4* ^+/+^ mice at 6 and 10 days PI (**Figure 4A**), while circulating NE levels gradually increased with infection but were not different between *Mcpt4^-/-^* and *Mcpt4* ^+/+^ mice at any time point (**Figure 4B**). In our network analyses (**Figure 7**), MPO was absent in *Mcpt4^-/-^* mice at 4 days PI and NE was indirectly correlated with blood 16S copies through plasma eotaxin. At 6 days PI, MPO and NE were positively correlated with blood 16S copies in *Mcpt4^-/-^* mice and negatively correlated in *Mcpt4* ^+/+^ mice. By 8 days PI, MPO was absent in *Mcpt4^-/-^* mice and NE was associated with blood 16S copies through RANTES/CCL5. MPO was not detected in the *Mcpt4* ^+/+^ network, previously, were observed significant increases in neutrophils by day 4 PI that declined to control levels by day 8 PI that could explain the lower levels of MPO at this time point (8). In ileum, NE was negatively associated with IL-12p40, TNF-α and RANTES/CCL5, and positively with plasma IL-9, IFN-γ and IgE. By 10 days PI in *Mcpt4^-/-^* mice, NE was positively correlated with IL-17, which was negatively correlated with parasitemia, while MPO in *Mcpt4* ^+/+^ mice was positively correlated with parasitemia. These results suggest very different roles of neutrophils in bacterial clearance and potential effects on parasitemia in *Mcpt4^-/-^* and *Mcpt4* ^+/+^ mice, perhaps through different activation mechanisms. Reactive oxygen species (ROS)-dependent signaling activation of neutrophil NETosis in response to microbial infections is well established (44). However, NET formation can occur through different signaling pathways depending on the stimulus (45), and NE and MPO have different functions and mechanisms of action. For example, MPO can inactivate NE through HOCl but not H_2_O_2_ (46). Mcpt4 has been associated with control of neutrophil extravasation and activation and with limiting associated damage in renal ischemia reperfusion injury (47). The distinct correlations of NE with bacterial 16S copies as well as the differences in MPO expression between the *Mcpt4^-/-^* and *Mcpt4* ^+/+^ mice suggest a novel role for Mcpt4 in neutrophil regulation in the context of malaria.

There is evidence in human malaria that an antibody response to gametocyte surface proteins can influence transmission to mosquitoes (27). It also has been suggested that enhanced transmission can be associated with low antibody titers and low inherent gametocyte infectivity (i.e. low gametocyte densities) (48–50). Relative to *Mcpt4* ^+/+^ mice, we observed modestly but significantly higher parasite transmission from *Mcpt4^-/-^* mice to *A. stephensi*. This was not reflected in differences in exflagellation, gametocytemia or parasitemia in *Mcpt4^-/-^* versus *Mcpt4* ^+/+^ mice. Transmission success has also been associated with male to female gametocyte ratio (50, 51) and gametocyte maturation (52). Moreover, although the effects of cellular immune responses to sexual stages and associated changes in transmission to mosquitoes is not clear (53), CD4 + T cells have been shown to respond to gametocyte antigens (54, 55). In the mosquito host, ingested mammalian blood factors can significantly and profoundly affect transmission success of both mouse and human malaria parasites to *A. stephensi*, independently of patterns of gametocytemia and exflagellation (56–59). While it is clear that Mcpt4-dependent host responses can impact parasite transmission to the mosquito host, additional studies are required to define mechanisms that may affect parasite development and maturation in both mammalian and mosquito hosts.

In conclusion, our studies show that Mcpt4 plays a role in sustaining *P. y. yoelii* 17XNL infection by promoting a type-2 immune response that concordantly protects intestinal epithelial barrier integrity and limits the systemic response to bacteremia. One possible explanation is that iRBC sequestration in the intestine (60), which is well known in falciparum malaria (11) and also reported by us for *P. yoelii* infection (60), triggers an early inflammatory response that induces local tissue damage and bacterial translocation. This in turn could induce MC activation and degranulation to control the immune response and initially protect intestinal barrier integrity, thereby delaying the immune response to the parasite and allowing parasite replication to proceed. Several studies indicate that MC proteases can inactivate pro-inflammatory mediators *via* degradation (16, 18, 28, 39), thereby restricting the early pro-inflammatory response to the parasite. This study provides novel findings regarding the contribution of Mcpt4 in promoting acute malaria while concordantly regulating intestinal barrier integrity and parasite transmission.

## Data Availability Statement

The original contributions presented in the study are included in the article/**Supplementary Material**. Further inquiries can be directed to the corresponding author.

## Ethics Statement

The animal study was reviewed and approved by Institutional Animal Care and Use Committee, University of Idaho.

## Author Contributions

SL contributed to conception, design of the study and funding acquisition. NC coordinated the research activities, organized the data, performed data analyses, and wrote the first draft of the manuscript. NC, ED, CL, GH, DW, AB, JS and LH contributed to performing the experiments and data collection. SL and JW wrote sections of the manuscript. MÅ provided the Mcpt4-/- mutant mouse line. All authors read the manuscript, contributed to revision and approved the submitted version.

## Funding

This work was funded by NIH NIAID RO1 AI131609 to SL. The funders had no role in study design, data collection and interpretation, or the decision to submit the work for publication.

## Conflict of Interest

The authors declare that the research was conducted in the absence of any commercial or financial relationships that could be construed as a potential conflict of interest.

## Publisher’s Note

All claims expressed in this article are solely those of the authors and do not necessarily represent those of their affiliated organizations, or those of the publisher, the editors and the reviewers. Any product that may be evaluated in this article, or claim that may be made by its manufacturer, is not guaranteed or endorsed by the publisher.
