# Supplementary material for: Mast Cell Chymase/Mcpt4 Suppresses the Host Immune Response to Plasmodium yoelii, Limits Malaria-Associated Disruption of Intestinal Barrier Integrity and Reduces Parasite Transmission to Anopheles stephensi
Source: Front Immunol. 2022 Jan 27;13:801120. doi: 10.3389/fimmu.2022.801120 (PMC8829543; doi:10.3389/fimmu.2022.801120)
Supplement: Supplementary file 1 [file DataSheet_1.pdf]

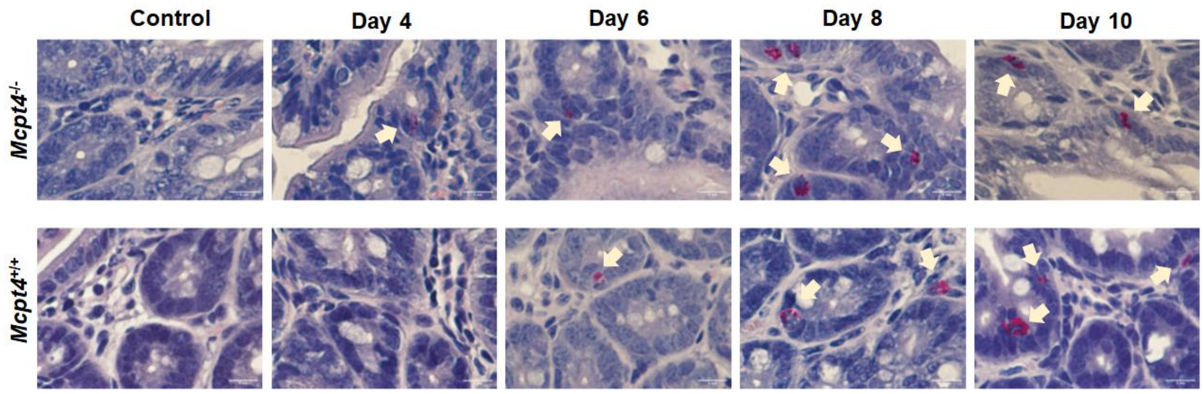

Supplementary Figure 1. Representative naphthol AS-D chloroacetate esterase (NASDCE) stained MCs (pink cells indicated by yellow arrows) in ileum of *Mcpt4*$^{-/-}$ (top) and *Mcpt4*$^{+/+}$ (bottom) mice in uninfected controls at indicated time points PI.

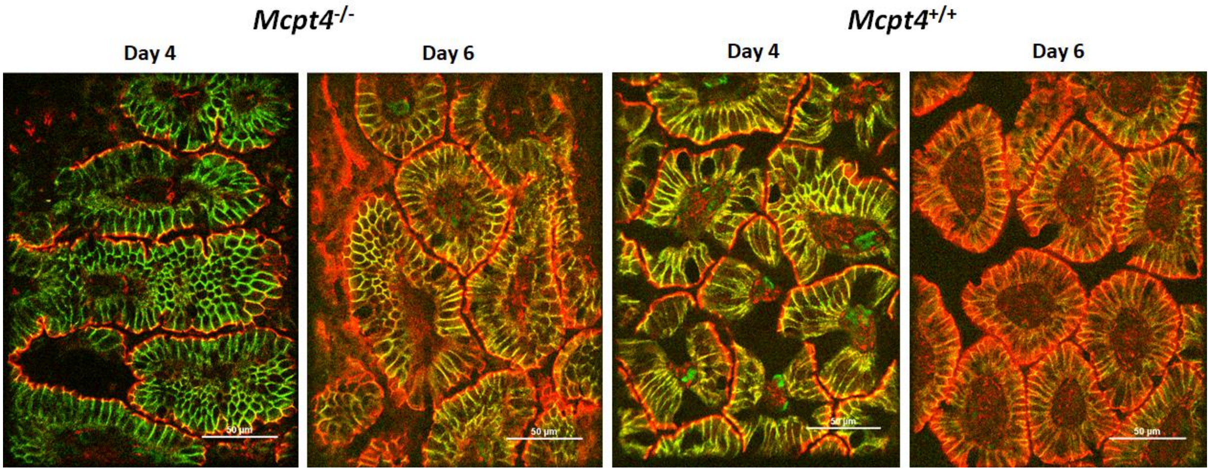

Supplementary Figure 2. Enlarged representative images of ileum tissue from *P. y. yoelii* 17XNL-infected *Mcpt4*$^{-/-}$ mice (left) and *Mcpt4*$^{+/+}$ mice (right) stained for E-cadherin (green) and ZO-1 (red) at days 4 and 6 post infection.

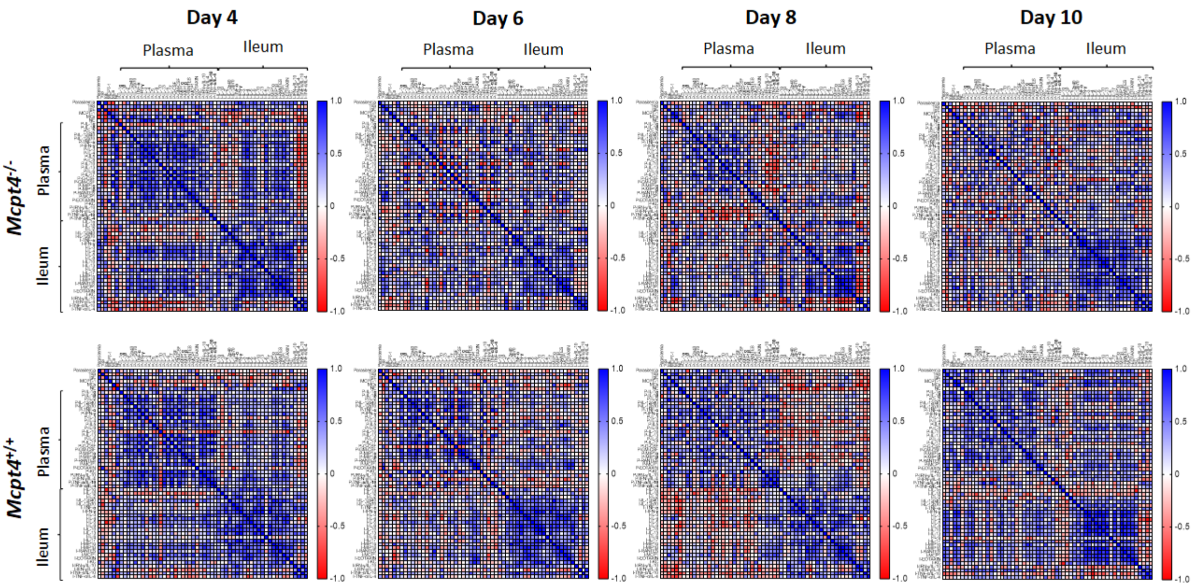

Supplementary Figure 3. Hierarchical clustering. Heat map representation of Spearman's correlations between parasitemia, blood 16S levels, ileal MC numbers, levels of plasma and ileal cytokines and chemokines, plasma IgE and plasma Mcpt1 for *P. y. yoelii* 17XNL-infected *Mcpt4*$^{-/-}$ mice (top) and *Mcpt4*$^{+/+}$ mice (bottom) at 4, 6, 8 and 10 days PI. The heat map colors correspond to correlations grading from 1 (positive correlation, blue), no correlation (white) to -1 (negative correlation, red). P values ≤ 0.05 were considered significant.
